# China’s health service collaboration in the Guangdong-Hong Kong-Macao Greater Bay Area: barriers and next steps

**DOI:** 10.3389/fpubh.2025.1442328

**Published:** 2025-05-27

**Authors:** Weidong Wu

**Affiliations:** School of Humanities, Jinan University, Zhuhai, Guangdong, China

**Keywords:** collaboration, health service, migrant, the Guangdong-Hong Kong-Macao Greater Bay Area, China

## Abstract

Health service collaboration in the Guangdong-Hong Kong-Macao Greater Bay Area is crucial in addressing the growing healthcare demands driven by increasing migration and economic integration. This study highlights the significance of such collaboration in enhancing cross-border medical services and responding to the region’s evolving public health needs. Despite its potential benefits, several challenges impede effective collaboration, including the lack of mutual recognition of medical qualifications, limited interconnectivity of health information systems, and divergent regulatory frameworks that complicate service delivery across the regions. To mitigate these obstacles, the study advocates for the establishment of comprehensive regulatory alignment, which would ensure mutual recognition of healthcare professionals’ qualifications. Furthermore, the development of integrated platforms for information sharing and service regulation is essential for fostering effective cooperation in delivering health services.

## Introduction

Intergovernmental collaboration takes place among national, state, and local governments to accomplish shared objectives (1). In China, collaborative governance is a relatively recent development, and public management’s ability to handle such collaboration is just beginning to take form (2). The integration of health services within the Guangdong-Hong Kong-Macao Greater Bay Area holds strategic importance for addressing the region’s public health demands, especially considering its rapid socio-economic development.

The establishment of the Greater Bay Area aims to optimize and integrate critical production factors and foster regional connectivity, which in turn fuels cross-border migration. Given these dynamics, the seamless integration of healthcare services is vital for supporting the Greater Bay Area’s evolving demographic and economic landscape. Promoting collaboration in health service will enable the efficient allocation and flow of medical resources, foster integrated health service development, and strengthen collaborative responses to public health emergencies, such as the COVID-19 pandemic.

Despite ongoing efforts, the current collaboration between Guangdong, Hong Kong, and Macao remains primarily focused on health emergency information sharing and experience exchange. In 2019, a significant step forward was the signing of the Guangdong-Hong Kong-Macao Greater Bay Area Health Cooperation Consensus, which outlines action plans for collaboration in areas such as medical technology, talent development, and disease prevention. The establishment of a joint prevention and control mechanism for infectious diseases marks a critical milestone, with joint meetings among health officials. This agreement enhances the region’s capacity for health emergency responses, infectious disease surveillance, and cross-border public health communication.

However, a comprehensive review of the regulatory alignment needed for cross-border health services across the Greater Bay Area is essential for advancing these collaborations. This paper seeks to explore the key regulatory challenges that hinder effective health service integration and offers actionable recommendations to address these issues.

## Why health service collaboration matters?

The primary beneficiaries of regional health service collaboration policies include cross-border workers, retirees seeking cross-border care, and individuals seeking specific medical services across borders. Health service collaboration in the Guangdong-Hong Kong-Macao Greater Bay Area particularly supports these groups by facilitating the efficient flow of medical resources and enhancing access to health services.

According to the Seventh National Population Census of China, as of November 2020, there were 371,380 residents from Hong Kong and 55,732 residents from Macao living in the 31 provinces of mainland China and registered in the census (3). This represents a significant increase compared to 2010, when the respective figures were 234,829 for Hong Kong and 21,201 for Macao, marking growths of 136,551 and 34,531, with respective increases of 58.1 and 162.9%. A substantial proportion of these residents are concentrated in mainland cities within the GBA, all located in Guangdong Province. In an analysis by the Hong Kong Census and Statistics Department, it was estimated that by mid-2015, approximately 516,000 Hong Kong residents were usually staying in Guangdong Province (4). Despite the disruptions caused by the COVID-19 pandemic, by the end of 2022, the number of Hong Kong residents typically staying in Guangdong Province remained high at 505,000, with 88,000 aged 65 or older (5). This figure reflects the growing trend of Hong Kong residents choosing to live and work in Guangdong, driven by the region’s economic integration, housing opportunities, and employment prospects within the Greater Bay Area.

The growing population of Hong Kong and Macao residents in Guangdong Province has led to a corresponding rise in their demand for health services in the region. An analysis of inpatient visits from residents of Hong Kong and Macao to Guangdong Province between 2018 and 2021 demonstrates a clear upward trend. In 2018, there were over 10,000 inpatient visits, with 8,037 from Hong Kong and 2,072 from Macao (6). By 2021, total visits had surged to 16,153, with 10,750 from Hong Kong and 5,403 from Macao (7). This data indicates a growth of approximately 61% overall, with Hong Kong residents increasing by about 33.8% and Macao residents experiencing a remarkable rise of approximately 160%. Between 2018 and 2021, the top five diseases leading to inpatient treatment for residents of Hong Kong and Macao shifted. Heart disease became the most common diagnosis by 2021, while benign tumors, malignant tumors, and cerebrovascular diseases remained consistently prevalent in both years. After the COVID-19 pandemic, cross-border medical consumption surged in the Greater Bay Area. Medical institutions in Shenzhen alone providing diagnostic and treatment services to 640,000 residents from Hong Kong and Macao in 2023 (8).

As the demand for health services among residents from Hong Kong and Macao continues to rise, the need for effective collaboration within the health service systems becomes increasingly critical. Firstly, expediting the collaboration of health service systems is a fundamental requirement to further promote labor mobility and optimize the allocation within the Guangdong-Hong Kong-Macao Greater Bay Area. With the development of the Greater Bay Area, the number of cross-region workers continues to increase. By the end of 2021, more than 85,100 Hong Kong and Macao residents were registered for employment in Guangdong Province (9). By the end of 2023, more than 120,000 mainland workers and 2,289 Hong Kong residents were employed in Macao (10).

The health care system is an essential component of modern social protection for workers, crucial for addressing health risks and safeguarding individual health rights. Moreover, caring for sick workers helps maintain a region’s human resource and strengthens the foundation for sustainable economic development (11). Although efficient transportation systems allow cross-region workers to return to their home locations for health services in a short period, obtaining health services directly in their destination area remains the preferable option (12), especially for routine and emergency health service needs (13). However, the absence of health service collaboration prevents migrant workers from accessing necessary health services in the destination area, potentially deterring migration behavior and restricting labor mobility (12). The disjointed health service systems between the Hong Kong, Macao and mainland cities in the Greater Bay Area has become significant barriers to labor migration (14).

Secondly, health service collaboration is essential for addressing health needs of cross-region retirees. Both Hong Kong and Macao are facing the challenge of rapid population aging, and cross-region retirement is increasingly considered a solution to this issue. Since 1997, the Hong Kong SAR government has paid growing attention to cross-region retirement, while the Macao SAR government has actively promoted cross-region retirement for Macao residents in Zhuhai’s Hengqin in recent years. In 2023, there were 89,000 Hong Kong residents aged 65 or older who typically resided in Guangdong Province (15). Given the relatively high demand for health service among the older adult, as well as their need for proximity to health service institutions, there is an urgent huge need for more accessible health services in their retirement residences. Exploring inter-regional arrangements for health services and improving the portability of health care across region are essential steps toward advancing cross-region retirement in the Guangdong-Hong Kong-Macao Greater Bay Area (16).

Lastly, health service collaboration plays a vital role in facilitating cross-border medical services. Due to limitations in local health services, some patients may choose to seek specific health services in other regions. Medical tourism, a relatively new form of tourism, has experienced rapid global growth since the late 1990s (11). In the mainland of China, cross-region medical treatment has been vigorously promoted in recent years to provide patients with more comprehensive health options. Factors such as long waiting times, limited health services, insufficient financial and human resources, and shifting demographics often prevent patients from receiving the desired health services in their place of residence (17). Furthermore, residents from economically developed regions may seek more affordable health services in other areas due to higher local medical costs (18).

The issue of inadequate medical resource supply in the Hong Kong and Macao has become increasingly severe, prompting residents to seek medical treatment in neighboring regions. In Hong Kong, the public health service system is under significant pressure, leading to long waiting times, and seeking medical treatment in the mainland of China has become an important way to alleviate the local healthcare burden (19). Despite being a highly developed metropolis, Hong Kong encounters substantial challenges in expanding its medical tourism sector, primarily due to insufficient capacity of its healthcare system (20). The rapid aging of the population, combined with the epidemiological shift toward chronic degenerative diseases, has placed tremendous strain on both health service provision and financing (19). For instance, statistics from the Hong Kong Hospital Authority reveal that between July 2023 and June 2024, the median waiting time for stable new case bookings at specialist outpatient clinics for ophthalmology was 55 weeks, with the longest wait extending up to 91 weeks (21).

Overcrowding and prolonged waiting times have become prevalent in Hong Kong’s hospitals (22). The city’s universal healthcare system has placed immense pressure on its public hospitals, leading to long waiting times, while private hospitals charge significantly higher fees compared to those in the mainland of China, making cross-region medical services an increasingly attractive option for residents (5).

Similarly, Macao’s healthcare system faces significant challenges in meeting growing demands, particularly in high-end medical services (23). As Macao’s solo public hospital, Conde S. Januário General Hospital reported an average waiting time of 3.4 weeks for initial specialist consultations in the first quarter of 2024 was 3.4 weeks (24). Due to limitations in case capacity and medical resources, certain medical conditions and surgeries, such as cardiovascular diseases, organ transplants, and gynecological radiotherapy, compel thousand Macao residents to seek appropriate treatment annually in Hong Kong and the mainland of China (5). At the same time, for mainland Chinese residents, Hong Kong remains one of several choices for outbound medical tourism (25).

## Barriers to progress

Like all organizational forms, collaborative governance is established within a specific context that integrates various combinations of economic, social, political, and cultural factors (2). The Guangdong-Hong Kong-Macao Greater Bay Area, operating under the “one country, two systems” framework, which results in diverse institutional regulations. Due to historical developments, the economic and social systems in Hong Kong, Macao and the mainland of China differ significantly from each other. Currently, the three regions exhibit considerable institutional differences in health service access, provision and regulation. Advancing cross-region health service collaboration requires overcoming significant barriers created by these institutional differences.

This study explores the major barriers to health service collaboration in the Greater Bay Area by focusing on four core components: medical qualifications, service provision, service payment, and service regulation.

### Medical qualifications

Medical qualifications form the foundation of healthcare service provision, and the physician licensing systems in Hong Kong, Macao, and mainland China operate independently of one another. These systems are not mutually recognized, reflecting the institutional differences between the three regions, which have developed under distinct legal frameworks. This disparity creates significant challenges, such as limitations on medical qualifications and the difficulties of mutual recognition of doctors’ professional rankings across regions (26). Mainland doctors, for instance, cannot practice in Hong Kong or Macao without first passing the local physician qualification exams. In emergencies requiring urgent transfer, mainland ambulances cannot cross the border, and mainland doctors are barred from practicing in Hong Kong; thus, patients must rely on private cross-border medical transport companies and only Hong Kong doctors, who are licensed in both regions, can accompany them (27).

While the mainland of China does issue short-term practice permits for Hong Kong and Macao doctors, their scope of practice remains restricted. For example, traditional Chinese medicine doctors from Macao, practicing at the First Affiliated Hospital of the Medical School of Macau University of Science and Technology in the Hengqin Guangdong-Macao In-Depth Cooperation Zone are authorized to prescribe medication but are prohibited from performing acupuncture. These restrictions prevent the full utilization, limiting their ability to deliver comprehensive patient care. Furthermore, the misalignment between mainland China’s professional title evaluation system and those of Hong Kong and Macao contributes to an exceptionally low passing rate for Hong Kong and Macao physicians, despite the opportunity for certification (13).

### Service provision

The interconnectivity and mutual recognition of medical information pose substantial challenges in cross-region service provision. As healthcare resources within the Greater Bay Area become more market-oriented, issues such as inconsistent technical standards and unclear responsibilities are emerging (28). The differences in hospital management systems, health service standards, and drug usage between Hong Kong, Macao, and the mainland of China, complicate the integration of medical information and services, which has, in turn, been challenging, reduced the effectiveness of pilot cross-border referral collaboration between healthcare institutions in Hong Kong and Guangdong (13). Furthermore, disparities in the management of medical records, healthcare data, and diagnostic test results have hindered the mutual recognition of records, resulting in additional time and costs for patients who must undergo repetitive tests and treatments, which adversely affect the cost-effectiveness of cross-region healthcare and diminish the overall patient experience (5).

At present, the interconnectivity of medical information is limited to specific hospitals in Shenzhen and applies only to patients from Hong Kong Hospital Authority who are temporarily in the mainland of China under specific circumstances. This limited scope underscores the broader challenge of achieving seamless medical information exchange across the three regions. Expanding this scope faces significant obstacles, particularly in areas such as patient privacy protection and managing medical risks. In Hong Kong, for example, the *Personal Data (Privacy) Ordinance* mandates that hospitals and clinics must provide patients with a Personal Information Collection Statement, explaining the purpose of data collection. Unauthorized disclosure of patient data without consent can result in severe penalties, including fines and imprisonment. Similarly, Macao’s *Personal Data Protection Law* classifies medical information as sensitive data. Mainland China made a significant step forward in 2021 by passing its first law specifically aimed at protecting personal information, the Personal Information Protection Law. Before this, there were no detailed provisions for safeguarding patient privacy in the context of the internet and big data (29). The new law will require time to be refined and its implementation strengthened to ensure its full effectiveness. This discrepancy may cause residents of Hong Kong and Macao to be hesitant in sharing personal information with mainland healthcare institutions, likely due to concerns over potential data breaches. There are still gaps in the cross-border transmission of medical data and cross-border healthcare services, particularly in terms of legal regulations, where the specific definitions of concepts are unclear, and the supervision of data flows remains singular and disorganized (30).

These challenges were exemplified during the COVID-19 pandemic, where privacy protection and legal concerns around the sharing of personal travel information hindered smooth border crossings between Guangdong and Hong Kong, a contrast to the more streamlined collaboration between Guangdong and Macao. The restrictions on interoperability between the Health Link system in Hong Kong and mainland hospitals negatively affected patient treatment and posed further obstacles to cross-region healthcare cooperation (27).

In addition to privacy issues, the mutual recognition of medical records also introduces medical risks. Medical records are critical for healthcare professionals’ analysis and diagnosis of a patient’s condition. Recognizing medical records from different hospitals involves certain risks, as healthcare professionals must review examination and diagnostic results from other institutions and use them as the basis for their diagnoses. This process is closely tied to the competency levels of healthcare professionals in different hospitals. Given the significant disparity in professional standards among healthcare institutions, promoting the mutual recognition of medical records is a formidable challenge. Even within the mainland of China, the mutual recognition of test results between hospitals of different levels has been slow to progress, and the added complexity of cross-region collaboration among the distinct health service systems in the Greater Bay Area further complicates this issue.

### Service payments

In terms of service payments, the lack of integration between health care systems significantly impedes the use of health services. Access to public health services is primarily administered within the framework of health care systems, which presents challenges to fostering cross-region collaboration. Therefore, integrating the healthcare systems across the Guangdong-Hong Kong-Macao Greater Bay Area is crucial for advancing cross-region health service collaboration.

The healthcare systems in the Greater Bay Area operate under varying structures. In Hong Kong and Macao, healthcare services are predominantly by government revenues, providing universal public healthcare to all residents. Conversely, mainland cities in the Greater Bay Area operate under a mixed system of social health insurance and universal health insurance, which includes individual contributions and links the level of benefits to these contributions (31). Importantly, the mainland healthcare system does not provide coverage for mainland residents seeking medical treatment in Hong Kong and Macao.

While Hong Kong and Macao offer partial financial support for their residents receiving medical care on the mainland—such as through medical vouchers—this support is limited in scope. For instance, the pilot program of the older adult healthcare voucher initiated by the Hong Kong SAR government at the University of Hong Kong-Shenzhen Hospital in 2015 only targeted individuals aged 65 and older, excluding other age groups from its benefits, while the scope of this support was quite restricted and faced challenges due to limited service coverage and funding (27). These limitations in the portability of healthcare benefits are key reasons why many older adult Hong Kong residents are reluctant to retire in mainland China or return to Hong Kong after initially moving to the mainland (32).

### Service regulation

Medical services often involve complex issues, including medical disputes and misconduct, making it critical to establish and enforce robust regulatory measures. In the context of the Guangdong-Hong Kong-Macao Greater Bay Area, the regulatory landscape is particularly complex due to the distinct legal frameworks governing healthcare in each region. Macao, as a Special Administrative Region of China, operates under a legal framework that allows for a substantial degree of autonomy in various domains, including its legal system (33). This is also the case in Hong Kong. The regulatory systems established by mainland China, Hong Kong, and Macao are influenced by their distinct legal traditions, which correspond to the socialist law system, common law system, and civil law system, respectively (34). Macao operates under a civil law system that emphasizes codified laws and statutory regulations, while Hong Kong follows a common law system, inherited from the British legal tradition, which places significant importance on judicial precedents and case law (35). Thus, within the “One Country, Two Systems” framework, the Greater Bay Area applies three different legal systems to healthcare regulation (36).

A critical issue in cross-region medical care is the legal jurisdiction associated with the location of health services and the patient’s place of residence, particularly when medical disputes arise. The jurisdiction in which a court handles a dispute can significantly influence the rights and interests of both plaintiffs and defendants (37). Patients often face challenges in navigating unfamiliar legal systems, complicating their ability to protect their rights effectively in the event of medical disputes (35). Factors such as jurisdictional differences, evidence collection procedures, and rights protection mechanisms are central to addressing these disputes. Without comprehensive regulatory cooperation across regions, the resolution of medical disputes becomes increasingly difficult, further constraining the development of cross-border healthcare services.

## Next steps

Achieving cross-region collaboration in healthcare services within the Greater Bay Area requires a fundamental alignment of health service regulations. In the area of medical qualifications, enhancing collaboration and exchanges among healthcare professionals is essential. As the direct providers of health services, healthcare professionals play a pivotal role in fostering social integration, bridging cultural differences, and developing solutions for cross-region healthcare delivery. Strengthening the exchange and training of medical talent between the mainland of China, Hong Kong, and Macao across various medical fields is essential for contributing to the construction of a “Healthy Bay Area” (38). This can be facilitated through the establishment of collaboration platforms for service provision, joint medical talent training programs, and the creation of cross-region clinical trial centers.

To ensure effective collaboration, it is important to identify the similarities and differences in the medical qualification systems of the three regions. A negotiation platform should be established to define the qualification requirements for medical professionals practicing across borders, thereby promoting the mutual recognition of medical qualifications. At the same time, potential challenges, such as the influx of foreign physicians, reductions in local doctors’ salaries, and the infringement of local professionals’ rights, must be proactively addressed. Special attention should be given to the concerns of healthcare providers from Hong Kong and Macao to ensure fair and equitable practices.

In terms of service provision, it is essential to advance collaborative mechanisms for medical referrals and emergency transfers. A key priority at this stage is the development of a robust system for cross-region sharing of health service information sharing, facilitating the interoperability and mutual recognition of medical records. Hong Kong University Shenzhen Hospital could serve as a pilot center to explore the standardization of disease classification codes, surgical procedure codes, and medical terminology. This would support the mutual recognition of medical records and streamline bidirectional referrals across regions. As this collaboration progresses, protecting patient privacy and preventing data breaches must be top priorities. Furthermore, the rights of medical professionals must also be safeguarded, with clear boundaries established for their responsibilities. This will help to protect them from legal disputes arising from cross-region collaborations, particularly in cases where interconnected medical systems may complicate accountability.

Establishing a cooperative mechanism for protecting healthcare rights across the Greater Bay Area is critical for reducing cross-region medical disputes and ensuring timely and effective responses when conflicts arise. Signing relevant cooperation agreements between Guangdong, Hong Kong, and Macao would offer a legal foundation for implementing arbitration interim measures ordered by courts or arbitration tribunals in all three regions (39). This framework could focus on resolving key issues, including jurisdiction, evidence collection, and rights protection, thus promoting a structured approach to managing medical disputes while safeguarding patient rights in cross-border healthcare services.

## Conclusion

This study underscores the critical need for health service collaboration within the Guangdong-Hong Kong-Macao Greater Bay Area, driven by increasing cross-region demand due to rising migration, an aging population, and deeper economic integration. The necessity for efficient cross-border healthcare services has become particularly apparent for migrant workers, retirees, and patients seeking specialized care. However, significant barriers impede effective collaboration, including the lack of mutual recognition of medical qualifications, disparities in healthcare service standards, and the absence of integrated health information systems across the three regions. These institutional differences have resulted in fragmented service delivery and inefficiencies that prevent patients from receiving timely and appropriate care.

To address these challenges, several key policy recommendations are proposed. First, promoting regulatory alignment is essential to ensure mutual recognition of healthcare qualifications and facilitate professional mobility. The development of robust platforms for sharing information and interoperability of medical records will also enhance service provision and reduce duplicative efforts. Furthermore, establishing a cooperative framework to safeguard patient privacy and manage cross-border medical risks is crucial for building trust in the system. These actions will not only improve access to healthcare but also strengthen the resilience and sustainability of the health services in the Greater Bay Area, positioning the region as a model for addressing cross-border healthcare challenges globally.

## References

[1] Cameron D. The structures of intergovernmental relations. Int Soc Sci J. (2002) 53:121–7. doi: 10.1111/1468-2451.00300

[2] Brown TL Gong T Jing J. Collaborative governance in mainland China and Hong Kong: introductory essay. Int Public Manag J. (2012) 4:393–404. doi: 10.1080/10967494.2012.761048

[3] National Bureau of Statistics of China (2021). The Seventh National Population Census Bulletin (No. 8) — Situation of Hong Kong, Macao, and Taiwan Residents and Foreign Nationals Registered in the Census. Available online at: https://www.stats.gov.cn/zt_18555/zdtjgz/zgrkpc/dqcrkpc/ggl/202302/t20230215_1904004.html (accessed October 2, 2024).

[4] Hong Kong Census and Statistics Department (2016). Estimation of Hong Kong Residents Usually Staying in Guangdong Province. Available online at: https://www.censtatd.gov.hk/en/data/stat_report/product/FA100274/att/B71606FA2016XXXXB0100.pdf (accessed Octorber 17, 2024).

[5] PwC China (2024). Integration and mutual benefit: A healthy Greater Bay Area. Available online at: https://www.pwccn.com/zh/research-and-insights/greater-bay-area/integration-healthy-sep2024.pdf (accessed October 17, 2024).

[6] Guangdong Provincial Health Commission Government Service Center (2019). Analysis of inpatient rates within counties of Guangdong Province in 2018. Available online at: https://www.gdhealth.net.cn/html/2019/tongjishuju1_0402/3853.html (accessed October 15, 2024).

[7] Guangdong Provincial Health Commission Government Service Center (2022). Analysis of inpatient rates within counties of Guangdong Province in 2021. Available online at: https://www.gdhealth.net.cn/html/2022/tongjishuju1_0512/4219.html (Accessed March 15, 2024).

[8] Pan YR. Cross-border medical boom promotes healthcare connectivity in the Greater Bay Area Securities Times (2024). Available at: http://www.stcn.com/article/detail/1252370.html (Accessed October 17, 2024).

[9] The Liaison Office of the Central People's Government in Macao (2021). The Greater Bay Area is Accelerating the Formation of a High-Quality Living Circle. Available online at: http://www.zlb.gov.cn/2022-06/01/c_1211652538.htm (accessed October 2, 2024).

[10] Macao Special Administrative Region Government, Statistics and Census Service (2024). Time Series Database. Available online at: https://www.dsec.gov.mo/en-US/Statistic/Database (accessed October 2, 2024).

[11] Arunanondchai BJ Fink C. Trade in health services in the ASEAN region. Health Promot Int. (2007) 21:59–66. doi: 10.1093/heapro/dal052, PMID: 17307958

[12] Werding M McLennan S. International portability of health-cost cover: mobility, insurance, and redistribution. Cesifo Working Paper No. 3952. (2012).

[13] Yang Q. Strategies for collaborative development of healthcare in the Guangdong-Hong Kong-Macao Greater Bay Area. Open J. (2020) 1:73–8. doi: 10.2139/ssrn.2159638

[14] Management Professional Group (2019). Research report on talent development environment in the Guangdong-Hong Kong-Macao Greater Bay Area. Available online at: https://www.mpgroup.cn/article_695.html (accessed January 17, 2024).

[15] Hong Kong Council of Social Service (2024). Research on the Current Situation and Challenges of Hong Kong Elders Aging in the Greater Bay Area. Available online at: https://www.hkcss.org.hk/upload/pra/GBAresearch_newsletter/240718_GBAresearch_brief_newsletter.pdf (accessed October 6, 2024).

[16] Hong Kong Macao J. Research on the impact of Guangdong-Hong Kong-Macao Greater Bay Area construction on cross-region elderly care: based on the perspective of welfare portability. J South China Univ Technol. (2020) 1:12–21. doi: 10.19366/j.cnki.1009-055X.2020.01.002

[17] Virani A Wellstead AM Howlett M. The north-south policy divide in transnational healthcare: a comparative review of policy research on medical tourism in source and destination countries. Glob Health. (2000) 16:1–15. doi: 10.1186/s12992-020-00566-3 PMC7178960 32321561

[18] Parekh J Jaffer A Bhanushali U Shukla S. Disintermediation in medical tourism through blockchain technology: an analysis using value-focused thinking approach. Inf Technol Tour. (2021) 23:69–96. doi: 10.1007/s40558-020-00180-4

[19] Gao Y Gong X Lin X. The role and opportunities of Hong Kong in the development of medical services in the Guangdong-Hong Kong-Macao Greater Bay Area. Hong Kong Macao Stud. (2019) 4:65–73+95–96.

[20] Heung V Kucukusta D Song H. Medical tourism development in Hong Kong: an assessment of the barriers. Tour Manag. (2011) 32:995–1005. doi: 10.1016/j.tourman.2010.08.012

[21] Hong Kong Hospital Authority (2024). Waiting Time for Stable New Case Booking at Specialist Out-patient Clinics. Available online at: https://www.ha.org.hk/haho/ho/sopc/dw_wait_ls.pdf (accessed October 7, 2024).

[22] Hong Kong Hospital Authority (2021). 2022 to 2027 Strategic Plan. Available online at: https://www.ha.org.hk/haho/ho/ap/HA_StrategicPlan2022-2027_TC_211216.pdf (accessed October 16, 2024).

[23] Wang Z Gao Q Lin Q. Many challenges in Macao’s medical reform. Chin Health. (2016) 7:109–10. doi: 10.15973/j.cnki.cn11-3708/d.2016.07.046

[24] Macao Health Bureau (2024). 150th anniversary series report of Conde S. Januário General Hospital-Proper Medical Care, Optimizing Services. Available online at: https://www.gov.mo/zh-hant/news/1068802/ (accessed October 7, 2024).

[25] Zhong X Chan C-S. Opportunities, challenges and implications of medical tourism development in Hong Kong. Int J Tour Res. (2024) 26:e2615. doi: 10.1002/jtr.2615

[26] Deng X Yao Z. Analysis of the efficiency of healthcare resource allocation in China's three major strategic regions. Mod Prev Med. (2022) 49:1631–5.

[27] Su QY Cai YJ Chen Y Yao RY. Breakthroughs and innovations in cross-border healthcare Services for the Elderly in Hong Kong under the Guangdong-Hong Kong-Macao Greater Bay Area context. J Guangzhou Inst Social. (2023) 3:83–8.

[28] Chen Y. Using digital technology to promote collaborative sharing of public services in the Guangdong-Hong Kong-Macao Greater Bay Area. China Adm Manag. (2023) 1:153–5.

[29] Guo J Zhang X. Analysis of the status of patient privacy breaches and privacy protection in China. J Med Inform. (2020) 2:21–8.

[30] Jin Y Ma C Ying Z. Research on obstacles, strategies, and technologies of cross-border livelihood data in the Greater Bay Area: taking cross-border health care data as an example. J Macau Stud. (2024) 1:159–72.

[31] Gu X. Toward quasi-universal public healthcare: organizational and institutional innovations in China’s basic medical security system. Soc Sci Res. (2017) 1:102–9.

[32] Hong Kong Legislative Council Secretariat Research Office (2018). Cross-border portability of welfare benefits in selected places (2017–2018). Available online at: https://www.legco.gov.hk/research-publications/chinese/1718in09-portability-of-welfare-benefits-in-selected-places-20180329-c.pdf (accessed October 18, 2024).

[33] Chen J. Coordination of legal conflicts under the "one country, two systems" framework. Int Econ Coop. (2007) 6:89–92.

[34] Liu K. Legal risks of combining internet healthcare and cross-border medical services: a case study of the Guangdong-Hong Kong-Macao Greater Bay Area. Chin Gen Pract. (2019) 22:3809–14.

[35] Feng ZH. The adjustment logic of inter-regional legal conflicts in public health in the Guangdong-Hong Kong-Macao Greater Bay Area. J Hong Kong Macao. (2023) 1:66–79+95.

[36] Wang MJ Hu PP Xing DN. Research on legal issues in cross-border healthcare. Jiangsu Health Serv Manag. (2017) 28:19–22.

[37] Raposo VL. Medical liability in Macao. In: Raposo VL Beran RG, editors. Medical liability in Asia and Australasia. Ius Gentium: Comparative perspectives on law and justice, vol. 94. Singapore: Springer (2022)

[38] Luo WY Sun B. Major practices, experiences, and prospects of coordinated healthcare development in the Guangdong-Hong Kong-Macao Greater Bay Area. Soft Sci Health. (2024) 38:29–34.

[39] Li Y. Research on enforcement of arbitral interim measures in the Guangdong-Hong Kong-Macao Greater Bay Area. J Macau Stud. (2023) 2:126–37.

